# Outcomes of Nivolumab‐Plus‐Ipilimumab in Metastatic Renal Cell Carcinoma: Second Interim Analysis of the J‐ENCORE Study

**DOI:** 10.1111/iju.70281

**Published:** 2025-11-25

**Authors:** Tomokazu Sazuka, Katsunori Tatsugami, Suguru Shirotake, Shuzo Hamamoto, Masahiro Nozawa, Kazuyuki Numakura, Atsushi Mizokami, Tsunenori Kondo, Sei Naito, Takashige Abe, Kojiro Ohba, Go Kimura, Shunta Onodera, Katsumi Yamaguchi, Hirotsugu Uemura

**Affiliations:** ^1^ Department of Urology, Graduate School of Medicine Chiba University Chiba Japan; ^2^ Department of Urology Kitakyushu Municipal Medical Center Fukuoka Japan; ^3^ Department of Uro‐Oncology Saitama Medical University International Medical Center Saitama Japan; ^4^ Department of Nephro‐Urology Nagoya City University Graduate School of Medical Sciences Aichi Japan; ^5^ Department of Urology Kindai University Faculty of Medicine Osaka Japan; ^6^ Department of Urology National Hospital Organization Osaka Minami Medical Center Osaka Japan; ^7^ Department of Urology Akita University Graduate School of Medicine Akita Japan; ^8^ Department of Renal and Urologic Surgery Asahikawa Medical University Hokkaido Japan; ^9^ Department of Integrative Cancer Therapy and Urology, Graduate School of Medical Science Kanazawa University Ishikawa Japan; ^10^ Department of Urology Tokyo Women's Medical University Adachi Medical Center Tokyo Japan; ^11^ Department of Urology Yamagata University Faculty of Medicine Yamagata Japan; ^12^ Department of Renal and Genitourinary Surgery Hokkaido University Graduate School of Medicine Hokkaido Japan; ^13^ Department of Urology Nagasaki University Graduate School of Biomedical Sciences Nagasaki Japan; ^14^ Department of Urology Nippon Medical School Hospital Tokyo Japan; ^15^ Ono Pharmaceutical Co. Ltd. Osaka Japan; ^16^ Bristol Myers Squibb Tokyo Japan

**Keywords:** ipilimumab, Japan, nivolumab, prospective study, renal cell carcinoma

## Abstract

**Objectives:**

J‐ENCORE is a multicenter prospective observational study in Japan involving advanced or metastatic renal cell carcinoma patients receiving nivolumab‐plus‐ipilimumab (NIVO+IPI) as first‐line treatment. The minimum 1‐year observation revealed the efficacy and safety of NIVO+IPI comparable to those in CheckMate 214, and evaluated patient characteristics across subgroups for response and early progression. This minimum 2‐year observation focuses on the post‐treatment status after NIVO+IPI discontinuation.

**Methods:**

The objective response rate (ORR), response duration, progression‐free survival (PFS), overall survival (OS), treatment‐related adverse events (TRAEs), treatment‐free status, and second‐line treatment were evaluated.

**Results:**

The study included 274 patients (median age, 68 years; 24.8% aged ≥ 75 years; 15.7% had an Eastern Cooperative Oncology Group performance status ≥ 2; 58.0%, intermediate risk; 42.0%, poor risk) from 37 sites, with a median follow‐up of 35.4 (range, 24.4–47.0) months. The ORR was 37.6%, with a median real‐world duration of response at 17.1 months; 30.2% had real‐world PFS ≥ 24 months, and the OS rate at 24 months was 67.2%. TRAEs of any grade, grade 3/4, and grade 5 occurred in 77.4%, 42.7%, and 1.1% of patients, respectively. Furthermore, 11.1% experienced late‐onset TRAEs 28–100 days after discontinuation. For patients discontinuing NIVO+IPI due to adverse events, median treatment‐free survival was 7.4 months; 34.9% had a treatment‐free interval ≥ 12 months. Second‐line treatment ORR was 22.0%, with cabozantinib as the most common choice.

**Conclusions:**

We determined the long‐term real‐world effectiveness and safety of NIVO+IPI, providing beneficial information on post‐treatment status.

**Trial Registration:**

UMIN Clinical Trials Registry number: UMIN000036772; ClinicalTrials.gov identifier: NCT04043975

**Abbreviations**
a/mRCC: advanced or metastatic renal cell carcinoma
AEs: adverse events
BOR: best overall response
ccRCC: clear cell RCC
CI: confidence interval
CR: complete response
DOR: duration of response
ECOG PS: Eastern Cooperative Oncology Group performance status
I/P: intermediate or poor
IMDC: International Metastatic Renal Cell Carcinoma Database Consortium
IO: immuno‐oncology
IQR: interquartile range
nccRCC: non‐clear cell RCC
NE: not estimated
NIVO: nivolumab
NIVO+IPI: nivolumab‐plus‐ipilimumab
NR: not reached
ORR: objective response rate
OS: overall survival
PD: progressive disease
PFS: progression‐free survival
PR: partial response
RECIST: Response Evaluation Criteria in Solid Tumors
rwDOR: real‐world duration of response
rwPFS: real‐world progression‐free survival
TFS: treatment‐free survival
TKIs: tyrosine kinase inhibitors
TRAEs: treatment‐related adverse events

## 1 Introduction

Dual immuno‐oncology (IO) combination therapy with nivolumab‐plus‐ipilimumab (NIVO+IPI) is recommended as first‐line treatment for patients with metastatic clear cell renal cell carcinoma (ccRCC), classified as intermediate or poor (I/P) risk by the International Metastatic Renal Cell Carcinoma Database Consortium (IMDC) [1, 2, 3]. In Japan, NIVO+IPI was approved in August 2018 for untreated advanced or metastatic renal cell carcinoma (a/mRCC) patients with IMDC I/P risk, based on the global phase 3 CheckMate 214 trial [4].

CheckMate 214 excluded patients with non‐clear cell RCC (nccRCC), Eastern Cooperative Oncology Group performance status (ECOG PS) ≥ 2, and brain metastases [4]. Evidence is needed for patients with this background in real‐world clinical settings. Most real‐world studies of NIVO+IPI have been conducted retrospectively [5, 6, 7, 8, 9, 10, 11, 12]. There is limited prospective real‐world data on the effectiveness and safety of NIVO+IPI for Japanese patients with a/mRCC.

IO drug‐based therapies demonstrated long‐term survival benefits and continued response after treatment discontinuation [13, 14]; however, they may cause persistent and late‐onset adverse events (AEs) [15], necessitating extended follow‐up. The management of RCC involves subsequent treatments, but comprehensive data on subsequent treatments after first‐line treatment remain limited for Japanese patients.

To fill these gaps in real‐world evidence, we conducted a multicenter prospective observational study, J‐ENCORE, examining the effectiveness and safety of NIVO+IPI in patients with previously untreated a/mRCC in Japanese real‐world settings. Our first interim analysis of J‐ENCORE, evaluating 274 patients over a minimum follow‐up of 12.4 months, demonstrated effectiveness and safety profiles comparable to those in the CheckMate 214 and evaluated patient characteristics across subgroups for response and early progression [16]. This second interim analysis extended the follow‐up to ≥ 24 months to demonstrate the long‐term effectiveness and safety of NIVO+IPI and investigate disease status and treatments after NIVO+IPI discontinuation in real‐world settings.

## 2 Material and Methods

### 2.1 Study Design and Patients

J‐ENCORE is a prospective, multicenter, observational study assessing the real‐world outcomes of NIVO+IPI in untreated patients with a/mRCC from 37 Japanese institutions (Table S1) [16]. Patients with a/mRCC who initiated NIVO+IPI as first‐line treatment from August 15, 2019 to August 14, 2021, and provided written consent were enrolled (Figure S1). The study included patients (1) aged ≥ 20 years, (2) with histologically confirmed RCC, (3) with I/P risk RCC according to the IMDC, (4) without previous systemic therapy for RCC, and (5) scheduled to initiate NIVO+IPI. Pregnant and/or lactating women were excluded (Figure S1). Data on demographics, clinical characteristics, health status, disease status, treatment, outcomes, and AEs were prospectively collected from the medical records between August 2019 and August 2024. J‐ENCORE includes two interim analyses (12 and 24 months) for exploratory purposes and a final analysis (36 months) after enrollment completion to evaluate the effectiveness and safety of NIVO+IPI. This second interim analysis used data until August 23, 2023, with ≥ 24 months of follow‐up (Figure S1). Safety data were entered into the electronic data capture system until March 13, 2024. The study was approved by the Institutional Review Board/Independent Ethics Committee of each study site and registered with the UMIN Clinical Trials Registry (UMIN000036772) and ClinicalTrials.gov (NCT04043975).

### 2.2 Objectives

The primary endpoint was objective response rate (ORR). Key secondary and exploratory endpoints are shown in Figure S1. This analysis examined the ORR and overall survival (OS) of first‐line treatment (NIVO+IPI), real‐world progression‐free survival (rwPFS), real‐world DOR (rwDOR), AE incidence, treatment patterns, treatment‐free survival (TFS), treatment‐free interval, and ORR and OS of second‐line treatment for patients with a/mRCC who received NIVO+IPI as first‐line systemic therapy in Japanese real‐world settings. To distinguish from clinical trial endpoints, we adopted the terms rwPFS and rwDOR, which were identical to those in our first interim report [16]. TFS was defined as the time from NIVO+IPI discontinuation to second‐line treatment initiation or death. Since AEs could serve as a barrier to subsequent treatments, this study focused on treatment discontinuations due to AEs to examine TFS and treatment‐free interval. Late‐onset treatment‐related AEs (TRAEs) were defined as those occurring ≥ 28 days after NIVO+IPI discontinuation. Safety monitoring continued for 100 days after NIVO+IPI discontinuation. Treatment patterns, treatment‐free interval, and best overall response (BOR) from NIVO+IPI initiation to last known alive date or death were visualized using a swimmer plot. Tumor assessments were conducted by investigators according to the Response Evaluation Criteria in Solid Tumors (RECIST) v1.1 methodology using scan images from clinical practice. Progressive disease (PD) was also determined by clinical progression. AE severity was assessed according to the Common Terminology Criteria for Adverse Events v4.0, Japanese Clinical Oncology Group version.

### 2.3 Statistical Analysis

Results are presented as medians (range or interquartile range [IQR]) for continuous variables and as numbers (percentages) for categorical variables. ORR was calculated with 95% confidence intervals (CIs) using the Clopper–Pearson method. Median rwDOR, rwPFS, OS, and TFS were estimated using the Kaplan–Meier method, with 95% CIs calculated using the Brookmeyer–Crowley method. Statistical analyses were conducted using SAS v9.4 (SAS Institute Inc., Cary, NC, USA).

## 3 Results

### 3.1 Patient Disposition and Baseline Characteristics

A total of 293 patients scheduled for NIVO+IPI and providing consent between September 2019 and August 2021 were enrolled. Of these, 274 patients who met the eligibility criteria were included in the analysis set [16]. The median follow‐up period was 35.4 (range, 24.4–47.0) months (Figure S1). Median age was 68 (range, 31–87) years. Among the 274 patients, 78.8% were male, 24.8% were aged ≥ 75 years, 15.7% had an ECOG PS ≥ 2, 58.0% were IMDC intermediate risk, 42.0% poor risk, 81.8% had ccRCC, and 2.6% had brain metastases (Table 1). During the follow‐up, 244 of 274 patients (89.1%) discontinued NIVO+IPI; among the 244 patients who discontinued NIVO+IPI, 116 (47.5%) discontinued due to PD and 86 (35.2%) due to AEs. More than half (132 of 244 patients, 54.1%) initiated second‐line treatment (Figure S2).

**TABLE 1:** Baseline characteristics of patients.

Characteristics	Overall
	*n* = 274
Male, *n* (%)	216 (78.8)
Median age, years (range)	68 (31–87)
≥ 75 years, *n* (%)	68 (24.8)
BMI^a^, kg/m^2^, *n* (%)	
< 18.5	31 (11.4)
≥ 18.5, < 25.0	167 (61.6)
≥ 25.0	73 (26.9)
Smoking status, *n* (%)	
Never	81 (29.6)
Past	106 (38.7)
Current	61 (22.3)
Unknown	26 (9.5)
ECOG PS, *n* (%)	
0	193 (70.4)
1	38 (13.9)
2	30 (10.9)
≥ 3	13 (4.7)
IMDC risk, *n* (%)	
Intermediate	159 (58.0)
Poor	115 (42.0)
Histology, *n* (%)	
ccRCC	224 (81.8)
nccRCC	50 (18.2)
With measurable disease^b^, *n* (%)	250 (91.2)
With primary tumor, *n* (%)	138 (50.4)
With sarcoma component, *n* (%)	32 (11.7)
Previous surgery, *n* (%)	141 (51.5)
Previous nephrectomy, *n* (%)	138 (50.4)
Radical nephrectomy, *n* (%)	106 (38.7)
Previous radiation therapy, *n* (%)	29 (10.6)
Number of metastatic organs, *n* (%)	
0	7 (2.6)
1	115 (42.0)
2	92 (33.6)
3	40 (14.6)
≥ 4	20 (7.3)
Site of metastasis, *n* (%)	
Lung	171 (62.4)
Lymph node	119 (43.4)
Bone	82 (29.9)
Liver	37 (13.5)
Brain	7 (2.6)

Abbreviations: BMI, body mass index; ccRCC, clear cell renal cell carcinoma; ECOG PS, Eastern Cooperative Oncology Group performance status; IMDC, International Metastatic Renal Cell Carcinoma Database Consortium; nccRCC, non‐clear cell renal cell carcinoma; RECIST, Response Evaluation Criteria in Solid Tumors.

^a^ Data were missing for three patients.

^b^ Measurable disease was assessed by investigators using RECIST v1.1.

### 3.2 Effectiveness

**FIGURE 1:**
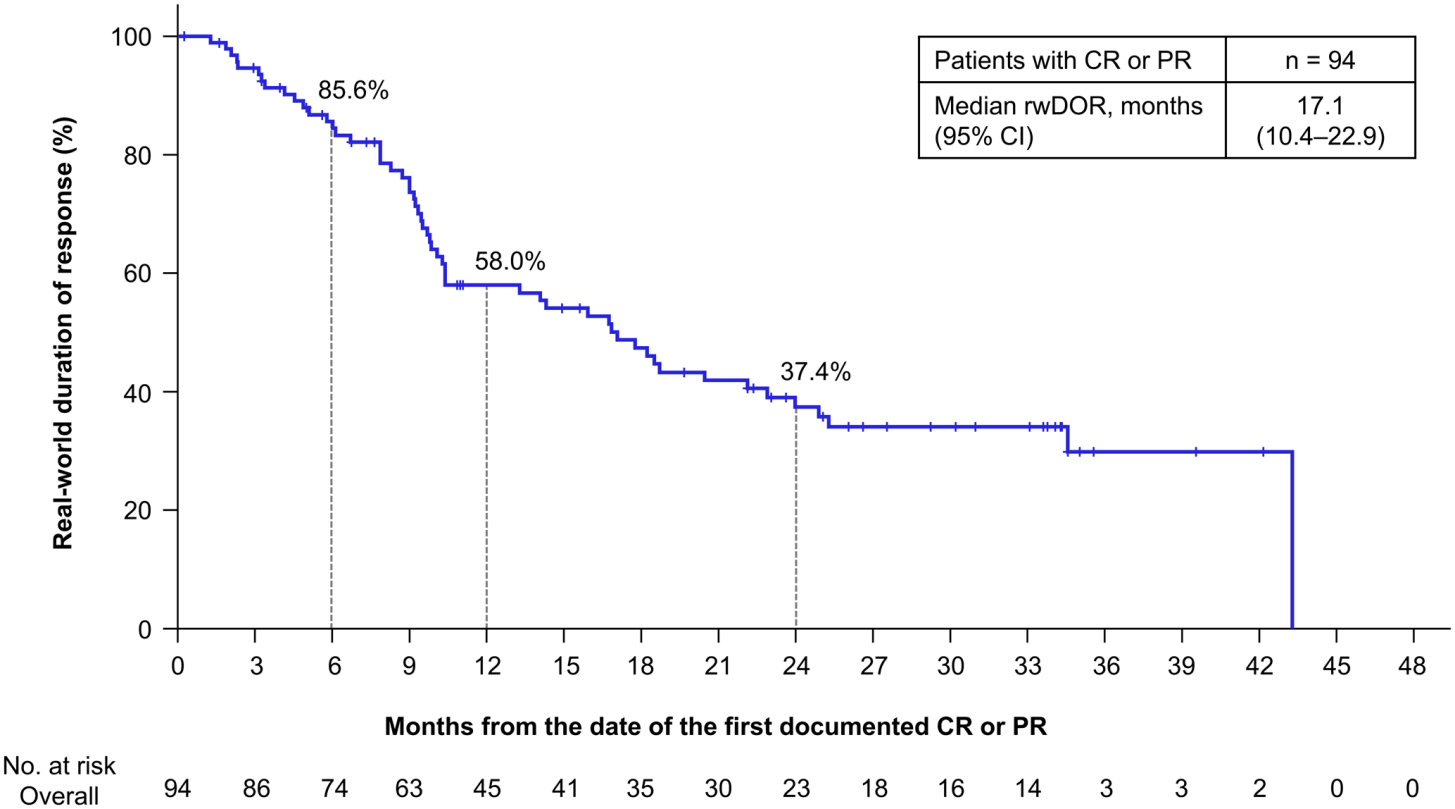
Real‐world duration of response. The rwDOR is shown in patients with CR or PR as the best response. rwDOR was defined as the time from the date of the first documented CR or PR after the initiation of NIVO+IPI to the date of the first documented PD or death from any cause. Radiological tumor assessments were conducted over time by investigators according to the RECIST v1.1 methodology using scan images obtained in clinical practice. PD was also determined by clinical progression. CI, confidence interval; CR, complete response; NIVO+IPI, nivolumab‐plus‐ipilimumab; PD, progressive disease; PR, partial response; RECIST, Response Evaluation Criteria in Solid Tumors; rwDOR, real‐world duration of response.

**FIGURE 2:**
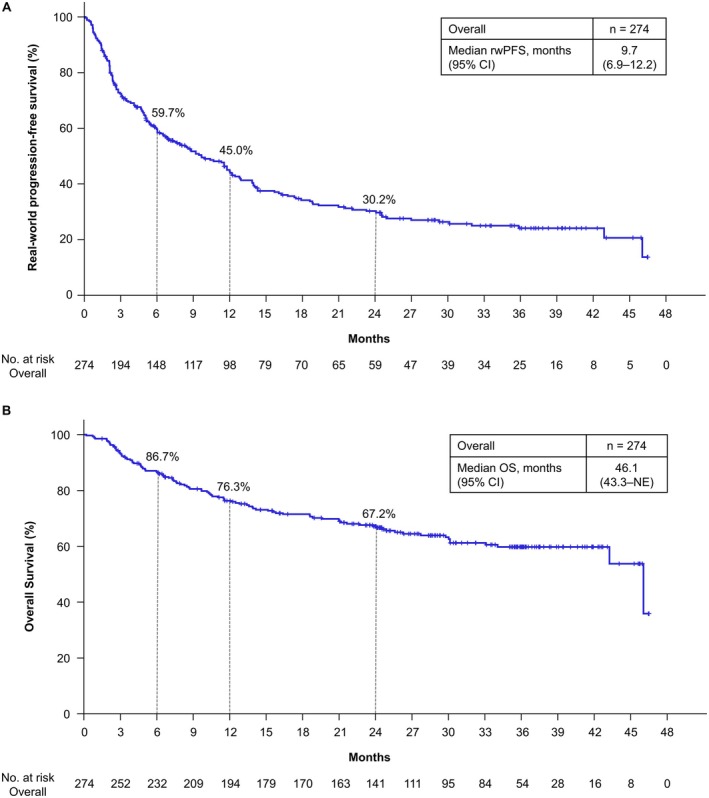
Real‐world progression‐free survival and overall survival. (A) rwPFS and (B) OS of all patients are shown. rwPFS was defined as the time from the date of initiation of NIVO+IPI to the date of the first documented PD or death from any cause. Radiological tumor assessments were conducted over time by investigators according to the RECIST v1.1 methodology using scan images obtained in clinical practice. PD was also determined by clinical progression. OS was defined as the time from the date of initiation of NIVO+IPI to the date of death from any cause. CI, confidence interval; NIVO+IPI, nivolumab‐plus‐ipilimumab; OS, overall survival; NE, not estimated; PD, progressive disease; RECIST, Response Evaluation Criteria in Solid Tumors; rwPFS, real‐world progression‐free survival.

The ORR among 250 patients with measurable disease was 37.6% (95% CI, 31.6%–43.9%; Table 2). Patients achieving a complete response (CR) or partial response (PR) had a median rwDOR of 17.1 (95% CI, 10.4–22.9) months (Figure 1). Among these responders, 58.0% maintained response for ≥ 12 months, and 37.4% maintained response for ≥ 24 months. During the 24‐month follow‐up, 185 of 274 patients (67.5%) experienced disease progression or died. For patients initiating NIVO+IPI, the median rwPFS was 9.7 (95% CI, 6.9–12.2) months, with 30.2% maintaining rwPFS for 24 months (Figure 2A). The median OS was 46.1 (95% CI, 43.3–not estimated [NE]) months, with 67.2% survival for 24 months (Figure 2B). The ORR by metastatic site and histological subtype (ccRCC, nccRCC) is shown in Table S2.

**TABLE 2:** Best overall response.

	Overall
	*n* = 250^a^
BOR, *n* (%)	
CR	16 (6.4)
PR	78 (31.2)
SD	70 (28.0)
PD	59 (23.6)
NE^b^	27 (10.8)
ORR, *n* (%)	94 (37.6)
95% CI	31.6–43.9
DCR, *n* (%)	164 (65.6)
95% CI	59.4–71.5
Median time to BOR, months (range)	
CR	5.2 (1.3–16.6)
PR	7.6 (0.1–36.7)

Abbreviations: BOR, best overall response; CI, confidence interval; CR, complete response; DCR, disease control rate; NE, not evaluable; ORR, objective response rate; PD, progressive disease; PR, partial response; RECIST, Response Evaluation Criteria in Solid Tumors; SD, stable disease.

^a^ Data were analyzed in 250 patients with measurable diseases, assessed by investigators according to RECIST v1.1. PD was also determined by clinical progression. The number of patients with measurable disease served as the denominator for calculating the BOR, ORR, and DCR.

^b^ NE included 24 patients for whom the assessment data were not available.

### 3.3 Safety

TRAEs of any grade occurred in 212 of 274 patients (77.4%), grade 3/4 TRAEs in 117 patients (42.7%), and grade 5 TRAEs in three patients (1.1%) (Table 3). The most common TRAEs of any grade were skin‐related events in 97 patients (35.4%), followed by endocrine‐related events in 84 patients (30.7%). Three patients (1.1%) died from treatment‐related hepatic, gastrointestinal, and cardiovascular disorders.

**TABLE 3:** Treatment‐related adverse events.

*n* (%)	Overall *n* = 274		
	Any grade	Grade 3/4	Grade 5
TRAEs	212 (77.4)	117 (42.7)	3 (1.1)
Skin	97 (35.4)	10 (3.6)	0 (0)
Endocrine	84 (30.7)	30 (10.9)	0 (0)
Hepatic	44 (16.1)	25 (9.1)	1 (0.4)
Respiratory	24 (8.8)	9 (3.3)	0 (0)
Diarrhea/colitis	21 (7.7)	11 (4.0)	0 (0)
Metabolism	18 (6.6)	16 (5.8)	0 (0)
Gastrointestinal	16 (5.8)	8 (2.9)	1 (0.4)
Renal	15 (5.5)	8 (2.9)	0 (0)
Nervous	15 (5.5)	5 (1.8)	0 (0)
Musculoskeletal	15 (5.5)	4 (1.5)	0 (0)
Cardiovascular	11 (4.0)	5 (1.8)	1 (0.4)
Hematotoxicity	8 (2.9)	8 (2.9)	0 (0)
Acute pancreatitis	4 (1.5)	3 (1.1)	0 (0)
Increased amylase and lipase levels	3 (1.1)	3 (1.1)	0 (0)
Other	52 (19.0)	11 (4.0)	0 (0)

*Note:* Grade was assessed by the investigators according to CTCAE v4.0.

Abbreviations: CTCAE, Common Terminology Criteria for Adverse Events; TRAE, treatment‐related adverse event.

Of 508 TRAEs occurring in 212 patients, 410 (80.7%) events were resolved (Table S3). TRAEs emerged at a median of 7.1 weeks after NIVO+IPI initiation and resolved within 8.0 weeks after onset (Table S3). Of the 274 patients, 164 (59.9%) received steroids for TRAE management. High‐dose steroids (≥ 40 mg per day of prednisone‐equivalent) were administered to 54 patients (19.7%), and 12 (4.4%) received steroid pulse treatment (≥ 500 mg per day of prednisone‐equivalent) (Table S4). Of 47 hepatic disorder‐related AEs, two (4.3%) received mycophenolate mofetil. No patients received infliximab to manage TRAEs.

**FIGURE 3:**
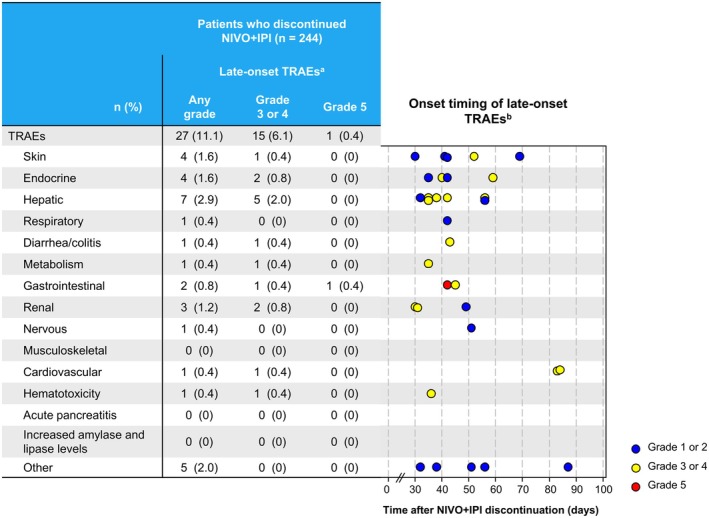
Late‐onset treatment‐related adverse events. ^a^The number of patients with late‐onset TRAEs is shown in the table on the left side. ^b^The timing of late‐onset TRAEs is shown in the scatter plot on the right side. When a patient experienced multiple TRAEs, all TRAEs were plotted. Each point in the scatter plot indicates the onset timing of late‐onset TRAE and its severity grade. The grade was assessed by the investigators according to CTCAE v4.0. CTCAE, Common Terminology Criteria for Adverse Events; NIVO+IPI, nivolumab‐plus‐ipilimumab; TRAEs, treatment‐related adverse events.

Among 244 patients who discontinued NIVO+IPI, 27 (11.1%) experienced late‐onset TRAEs of any grade, 15 (6.1%) experienced grade 3/4 TRAEs, and one (0.4%) experienced grade 5 gastrointestinal TRAE (Figure 3). Hepatic events were frequently observed among late‐onset TRAEs. These typically occurred 30–60 days after NIVO+IPI discontinuation. Grade 5 gastrointestinal TRAE was observed 42 days after discontinuation. Four late‐onset TRAEs occurred > 60 days after discontinuation in three patients, including one skin‐related event (grade 1 maculopapular rash at 69 days), two cardiovascular events occurring in one patient (grade 3 pulmonary embolism at 83 days, followed by grade 3 deep vein thrombosis at 84 days), and one other event (grade 2 myelodysplastic syndromes at 87 days).

### 3.4 TFS

**FIGURE 4:**
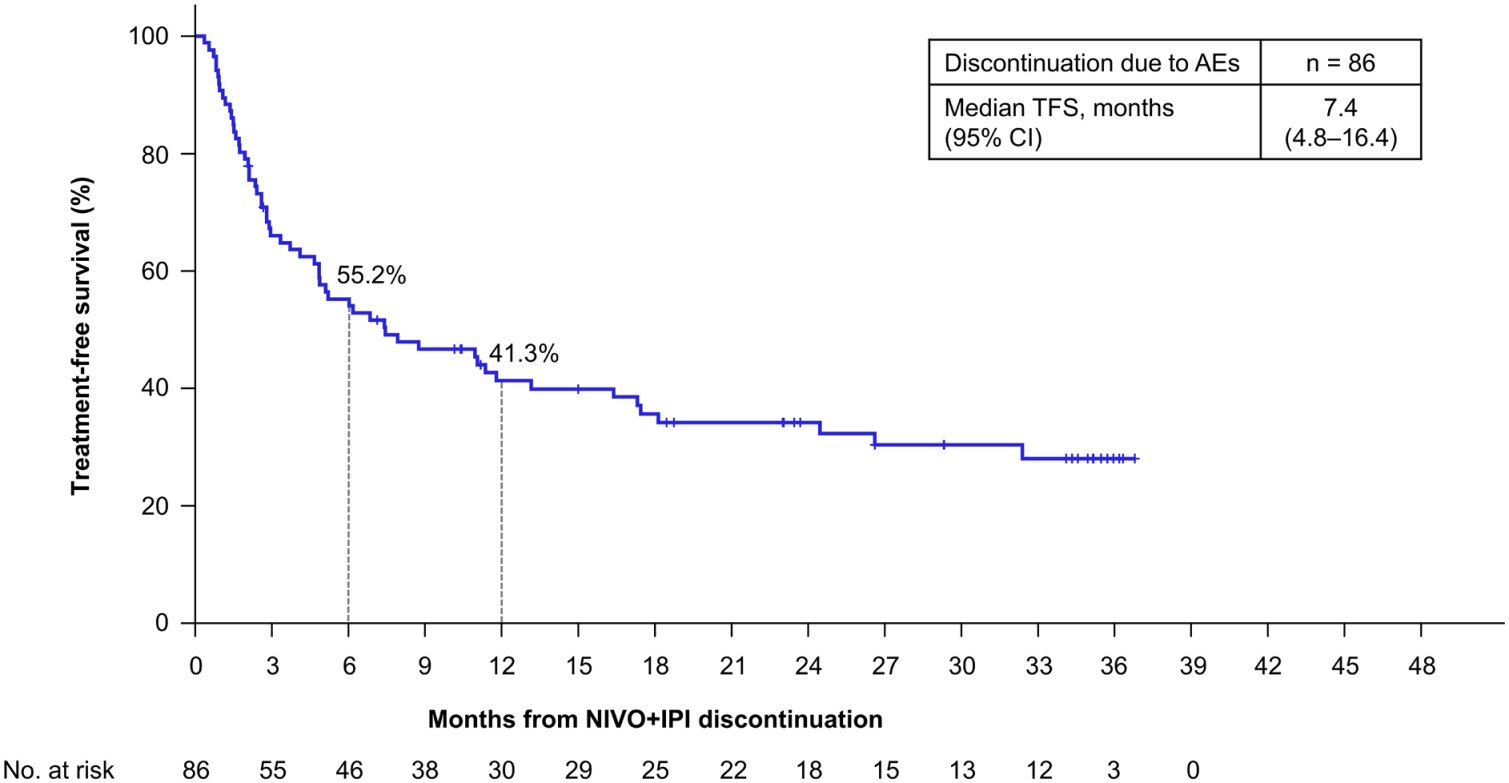
Treatment‐free survival after nivolumab‐plus‐ipilimumab discontinuation due to adverse events. TFS was defined as the time from NIVO+IPI discontinuation to the initiation of second‐line treatment or the date of death from any cause. AEs, adverse events; CI, confidence interval; NIVO+IPI, nivolumab‐plus‐ipilimumab; TFS, treatment‐free survival.

**FIGURE 5:**
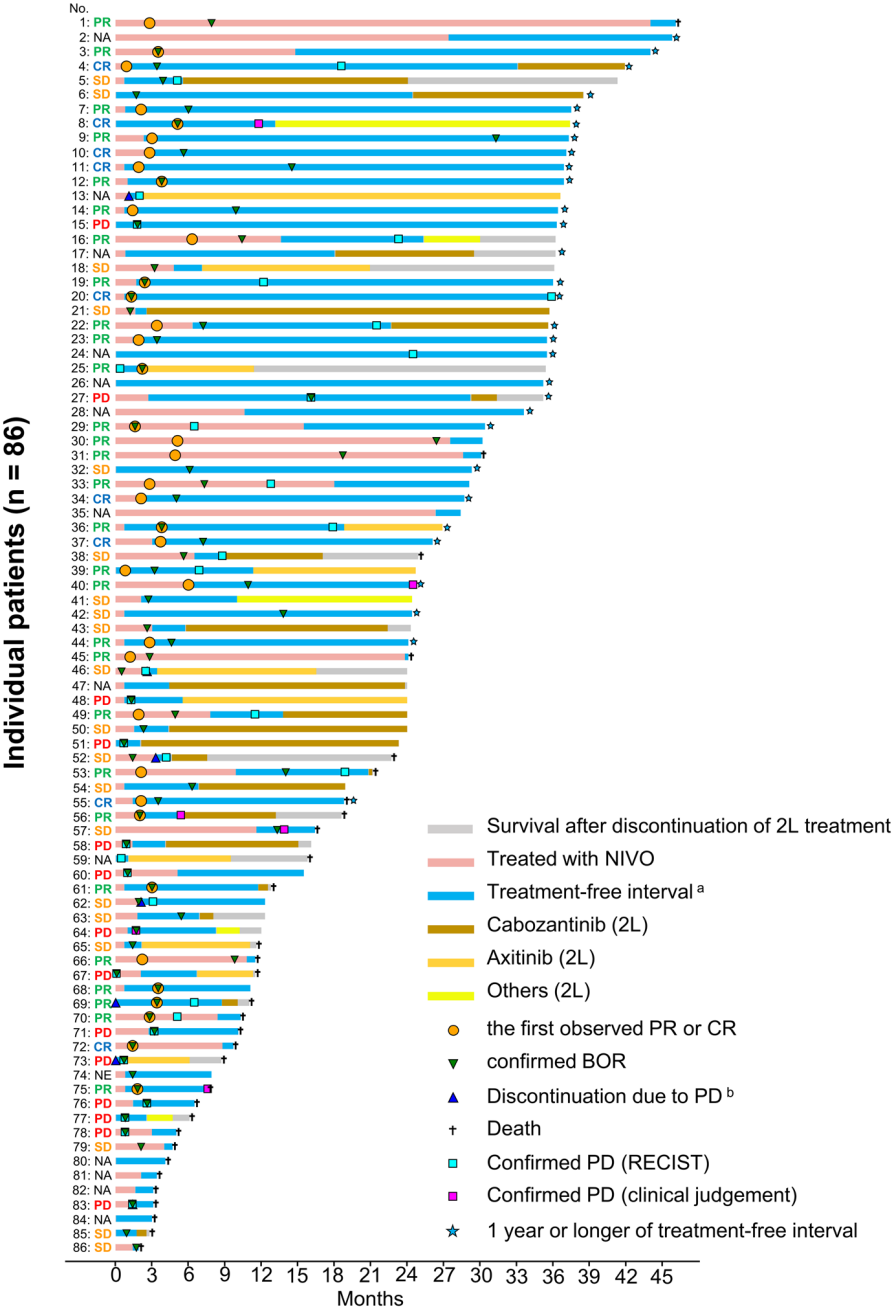
Swimmer plot for each patient who discontinued nivolumab‐plus‐ipilimumab due to adverse events. The swimmer plot illustrates the time course of the duration of NIVO treatment, treatment‐free interval, second‐line therapy, BOR, and survival from initiating NIVO+IPI to the last known alive date or the date of death for the 86 patients who discontinued NIVO+IPI due to AEs. Each bar represents one patient. Objective responses assessed by investigators per RECIST v1.1 are shown for each bar on the left side. Of the 13 patients with NA, nine had no measurable disease at the initiation of NIVO+IPI. ^a^Treatment‐free interval was defined as the time from NIVO+IPI discontinuation to the initiation of second‐line treatment or the date of death from any cause. ^b^Discontinuation due to both AEs and PD occurred in seven patients (shown as blue triangles). AEs, adverse events; BOR, best overall response; CR, complete response; NA, not assessed; NE, not estimated; NIVO, nivolumab; NIVO+IPI, nivolumab‐plus‐ipilimumab; PD, progressive disease; PR, partial response; RECIST, Response Evaluation Criteria in Solid Tumors; SD, stable disease; 2L, second‐line.

TFS and treatment‐free interval were evaluated for 86 patients who discontinued NIVO+IPI due to AEs. Their baseline characteristics were comparable to those of all patients (Table S5). The median TFS was 7.4 (95% CI, 4.8–16.4) months (Figure 4). A swimmer plot illustrates the duration of nivolumab (NIVO) treatment, treatment‐free interval, second‐line treatment, BOR, and survival for 86 patients who discontinued NIVO+IPI due to AEs. Among the 86 patients, 30 (34.9%) experienced a treatment‐free interval lasting ≥ 12 months after discontinuation, and 47 (54.7%) achieved a BOR, with 25 (29.1%) achieving CR or PR after discontinuation (Figure 5).

### 3.5 Second‐Line Treatment

**FIGURE 6:**
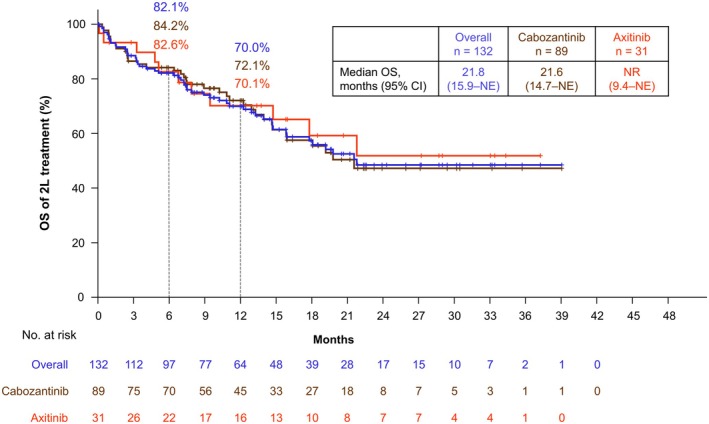
Overall survival after initiation of second‐line treatment. OS after initiation of 2L treatment is shown. OS was defined as the time from the date of the initiation of 2L treatment to the date of death from any cause. Other 2 L treatments, including pazopanib, sunitinib, nivolumab, and sorafenib (*n* = 12; Figure S2), could not be plotted because of low patient counts. CI, confidence interval; NE, not estimated; NR, not reached; OS, overall survival; 2L, second‐line.

Among the 132 patients receiving second‐line treatment after NIVO+IPI discontinuation, cabozantinib was most prescribed in 89 patients (67.4%), followed by axitinib in 31 patients (23.5%) (Figure S2). The median period of second‐line treatment was 210.0 (IQR, 59.0–379.0) days. The ORRs for second‐line treatment were 22.0% for all treatments, 25.8% for cabozantinib, and 19.4% for axitinib (Table 4). Baseline characteristics of the 132 patients receiving second‐line treatment were comparable to those of all patients (Table S6). Median OS after second‐line treatment initiation was 21.8 (95% CI, 15.9–NE) months for all treatments, 21.6 (95% CI, 14.7–NE) months for cabozantinib, and not reached (NR) (95% CI, 9.4–NE months) for axitinib (Figure 6).

**TABLE 4:** Best overall response of second‐line treatment after nivolumab‐plus‐ipilimumab.

	Initiating 2L treatment			
	All	Cabozantinib	Axitinib	Others^a^
	*n* = 132	*n* = 89	*n* = 31	*n* = 12
BOR^b^, *n* (%)				
CR	0 (0)	0 (0)	0 (0)	0 (0)
PR	29 (22.0)	23 (25.8)	6 (19.4)	0 (0)
SD	46 (34.8)	33 (37.1)	12 (38.7)	1 (8.3)
PD	14 (10.6)	8 (9.0)	3 (9.7)	3 (25.0)
NE^c^	43 (32.6)	25 (28.1)	10 (32.3)	8 (66.7)
ORR^b^, *n* (%)	29 (22.0)	23 (25.8)	6 (19.4)	0 (0)
95% CI	15.2–30.0	17.1–36.2	7.5–37.5	0.0–26.5
DCR^b^, *n* (%)	75 (56.8)	56 (62.9)	18 (58.1)	1 (8.3)
95% CI	47.9–65.4	52.0–72.9	39.1–75.5	0.2–38.5

*Note:* The median period of 2L treatment was 210.0 (IQR, 59.0–379.0) days.

Abbreviations: 2L, second‐line; BOR, best overall response; CI, confidence interval; CR, complete response; DCR, disease control rate; IQR, interquartile range; NE, not evaluable; ORR, objective response rate; PD, progressive disease; PR, partial response; RECIST, Response Evaluation Criteria in Solid Tumors; SD, stable disease.

^a^ Others include pazopanib, sunitinib, nivolumab, and sorafenib.

^b^ Data were assessed by investigators according to RECIST v1.1. PD was also determined by clinical progression. The number of patients who initiated 2L treatment was used as the denominator to calculate the BOR, ORR, and DCR, because data on their measurable disease were not collected at the initiation of 2L treatment.

^c^ NE included patients for whom disease assessment was not conducted.

## 4 Discussion

The effectiveness of NIVO+IPI for patients with a/mRCC with I/P risk in J‐ENCORE with 24 months of minimum follow‐up was almost comparable to that in CheckMate 214 with a median follow‐up of 32.4 months [17]. In CheckMate 214, the ORR was 42%, the 24‐month OS was 66%, and the median PFS was 8.2 months, with a 24‐month PFS rate of 30%. Previous retrospective real‐world data in Japan showed the ORR of 41.5%–57.0%; the median OS between 26.9 months and NR, with a 24‐month OS of 55%–67.4%; and the median rwPFS between 9 months and NR, with a 24‐month rwPFS of 38.5%–44.5% [5, 6, 7, 8, 9, 10, 11, 12]. To the best of our knowledge, J‐ENCORE is the first prospective real‐world study demonstrating the effectiveness comparable to the efficacy in CheckMate 214, supporting previous retrospective findings.

Among responders to NIVO+IPI, the median rwDOR was 17.1 months in J‐ENCORE with 24 months of minimum follow‐up, whereas the median DOR in CheckMate 214 was NR for 32.4 months of median follow‐up [17] and 82.8 months for 8 years of minimum follow‐up [14]. The shorter median DOR in J‐ENCORE could be attributed to earlier patient withdrawal after achieving a CR or PR in real‐world settings. This discrepancy may be affected by two factors. First, CheckMate 214 required confirmation, calculating DOR for patients with confirmed CR or PR diagnosed twice or more at ≥ 4 weeks intervals, while rwDOR in J‐ENCORE included patients achieving CR or PR once, including even those who experienced PD shortly after an initial response. Second, J‐ENCORE had more poor‐prognosis patients, including those without nephrectomy and with ECOG PS ≥ 2. The nephrectomy rate was lower in J‐ENCORE (50.5%) than in CheckMate 214 (80%) [4]. A post hoc analysis of CheckMate 214 showed that the median DOR of NIVO+IPI for non‐nephrectomy patients was 20.5 months [18], shorter than 82.8 months for the intent‐to‐treatment population [14]. When comparing ECOG PS, the median DOR of NIVO+IPI for metastatic non‐small cell lung cancer was 15.5 months in patients with ECOG PS 2 versus 27.6 months in patients with ECOG PS 0–1 [19]. Despite short rwDOR, the effectiveness of NIVO+IPI needs longer evaluation, as CheckMate 214 showed PFS and DOR plateaued at 2 years, suggesting sustained effects in responders. This emphasizes the evaluation of not only the median value but also long‐term trends. Since similar trends were observed, further extended follow‐up is required in J‐ENCORE.

During the extended follow‐up of > 24 months, no new TRAEs or increased frequencies were reported compared with the results of the previous 12‐month follow‐up [16], indicating no additional safety concerns for NIVO+IPI. Since the previous report [16], TRAEs were identified in seven patients, affecting the skin in three patients, the endocrine system in one, the respiratory system in one, the renal system in one, and others in one. High‐dose steroids were newly administered in one patient.

The mechanism of action of IO therapy can result in TRAEs after discontinuation or completion of treatment [20]. This study defined late‐onset TRAEs as those occurring 28 days or later after NIVO+IPI discontinuation, with follow‐up until 100 days after discontinuation. The 28‐day threshold was based on the longest dosing interval of NIVO to exclude TRAEs that caused NIVO+IPI discontinuation. Most late‐onset TRAEs occurred within 60 days after discontinuing NIVO+IPI, with four TRAEs occurring > 60 days after discontinuation. IO‐associated membranous nephropathy cases were detected 4 months or later after treatment discontinuation [21]. While the incidence of late‐onset TRAEs was low, continuously monitoring patients treated with NIVO+IPI is crucial even after discontinuation.

The effects of IO therapy can persist even after discontinuation, and TFS is a novel outcome measure for patients who discontinued IO therapy [22]. Since treatment discontinuation may cause anxiety, clarifying the actual treatment status after NIVO+IPI discontinuation in clinical practice is necessary. Of the 274 patients, 244 (89.1%) discontinued NIVO+IPI. Among those who discontinued NIVO+IPI, 116 (47.5%) discontinued due to PD, and 86 (35.2%) due to AEs (Figure S2). We focused on TFS for patients who discontinued NIVO+IPI due to AEs because in CheckMate 214, TFS was short in PD discontinuation cases but most durable in AE discontinuation cases [23]. Among 86 patients who discontinued NIVO+IPI due to AEs, the median TFS was 7.4 months, and 30 patients (34.9%) experienced a treatment‐free interval of ≥ 12 months. To the best of our knowledge, this is the first report on TFS after NIVO+IPI discontinuation due to AEs obtained from real‐world data. More than half of patients achieved their BOR after discontinuing NIVO+IPI due to AEs, with almost half experiencing CR or PR, indicating continued tumor reduction after discontinuation. This suggests that tumor reduction may continue even after treatment discontinuation, and that patients who discontinue NIVO+IPI due to AEs may still derive meaningful clinical benefit. However, when interpreting treatment‐free interval and TFS, it should be noted that patients who discontinued NIVO+IPI due to AEs may include those not receiving tumor treatment or still treating AEs.

Patients receiving first‐line dual IO combination therapy had not been exposed to tyrosine kinase inhibitors (TKIs). Many TKI‐naïve patients receive vascular endothelial growth factor TKIs as second‐line treatment after dual IO combination therapy [24, 25, 26, 27, 28, 29]. Compared to other real‐world data (ORR, 31%–44.8%; OS, 18 months–NR) [24, 25, 26, 28, 29], this study showed lower results (ORR, 22.0%; OS, 21.8 months). However, 32.6% of patients initiating second‐line treatment had no tumor assessment during the second‐line treatment, and a quarter were followed up for 59.0 days or less. Therefore, second‐line treatment outcomes could be underestimated, requiring further evaluation over extended follow‐up.

This study had limitations. First, the observational nature carries a potential selection bias because the use of NIVO+IPI was at the physician's discretion. Second, decisions regarding treatment continuation were determined by the physician. Third, the effectiveness and safety evaluations relied on physician‐determined examinations without independent central review of ORR and PFS. The follow‐up time may be inadequate for assessing NIVO+IPI in clinical practice compared to the 8‐year follow‐up data in CheckMate 214 [14]. As this was an interim analysis for exploratory purposes, the results should be considered preliminary. Lastly, baseline characteristics for second‐line treatment were not collected, which prevented a direct comparison of the efficacy of second‐line therapies.

This study demonstrated the effectiveness and safety of NIVO+IPI in treating patients with advanced RCC in Japanese clinical settings. Our results were comparable to those of CheckMate 214, except for shorter median rwDOR in J‐ENCORE. The effects persisted after discontinuation, with some patients achieving a 12‐month treatment‐free interval, while others experienced late‐onset AEs. Continuous monitoring after NIVO+IPI discontinuation and proper AE management are recommended to maximize the therapeutic benefits.

## Author Contributions

**Tomokazu Sazuka:** resources, writing – review and editing, conceptualization, investigation, supervision, visualization, writing – original draft. **Katsunori Tatsugami:** resources, writing – review and editing, conceptualization, investigation, writing – original draft. **Suguru Shirotake:** resources, writing – review and editing, investigation. **Shuzo Hamamoto:** resources, writing – review and editing, investigation. **Masahiro Nozawa:** resources, writing – review and editing, investigation. **Kazuyuki Numakura:** resources, writing – review and editing, investigation. **Atsushi Mizokami:** resources, writing – review and editing, investigation. **Tsunenori Kondo:** resources, writing – review and editing, investigation. **Sei Naito:** resources, writing – review and editing, investigation. **Takashige Abe:** resources, writing – review and editing, investigation. **Kojiro Ohba:** resources, writing – review and editing, investigation, writing – original draft. **Go Kimura:** resources, writing – review and editing, investigation. **Shunta Onodera:** resources, writing – review and editing, funding acquisition, methodology, visualization, writing – original draft, project administration. **Katsumi Yamaguchi:** resources, writing – review and editing, funding acquisition, methodology, visualization, writing – original draft, project administration. **Hirotsugu Uemura:** resources, writing – review and editing, conceptualization, investigation, methodology, supervision, visualization.

## Ethics Statement

The Ethics Committee of Kindai University Hospital approved the protocol (31‐061). This study was approved by the ethics committee of each hospital and was conducted in compliance with the Ethical Guidelines and the Declaration of Helsinki.

## Consent

Written informed consent was obtained from each patient before data collection.

## Conflicts of Interest

Tomokazu Sazuka and Suguru Shirotake received honoraria or others from Bristol Myers Squibb and Ono Pharmaceutical Co. Ltd. Shuzo Hamamoto received research funds from Boston Scientific Corporation and Daiwa Securities Foundation. Tsunenori Kondo received honoraria or others from Takeda Pharmaceutical Company Limited, MSD K.K., and Eisai Co. Ltd. Sei Naito received honoraria or others from Bristol Myers Squibb. Kojiro Ohba received honoraria or others from Bristol Myers Squibb, Ono Pharmaceutical Co. Ltd., and MSD K.K. Go Kimura received honoraria or others from Merck Biopharma Co. Ltd. and Bristol Myers Squibb. Shunta Onodera is an employee of Ono Pharmaceutical Co. Ltd. Katsumi Yamaguchi is an employee of Bristol Myers Squibb. Hirotsugu Uemura received honoraria or others from Bristol Myers Squibb, Ono Pharmaceutical Co. Ltd., and Takeda Pharmaceutical Company Limited and research funds from Ono Pharmaceutical Co. Ltd., Takeda Pharmaceutical Company Limited, Astellas Pharma Inc., and Pfizer Japan Inc. The other authors have no conflicts of interest to disclose. Kazuyuki Numakura, Atsushi Mizokami, Sei Naito, and Takashige Abe are the Editorial Board members of the International Journal of Urology and the co‐authors of this article. To minimize bias, they were excluded from all editorial decision‐making related to the acceptance of this article for publication.

## Supporting information


**Table S1:** List of investigators.
**Table S2:** Best overall response by metastatic site and histology.
**Table S3:** Time to onset and resolution of treatment‐related adverse events.
**Table S4:** Patterns of treatment‐related adverse event management.
**Table S5:** Baseline characteristics of patients who discontinued nivolumab‐plus‐ipilimumab due to AEs.
**Table S6:** Baseline characteristics of patients who initiated second‐line treatment.
**Figure S1:** Study design.
**Figure S2:** Patient disposition.

## Data Availability

Bristol Myers Squibb's policy on data sharing may be found at https://www.bms.com/researchers‐and‐partners/independent‐research/data‐sharing‐request‐process.html.
