# Supplementary material for: Outcomes of Nivolumab‐Plus‐Ipilimumab in Metastatic Renal Cell Carcinoma: Second Interim Analysis of the J‐ENCORE Study
Source: Int J Urol. 2025 Nov 25;33(1):e70281. doi: 10.1111/iju.70281 (PMC12835681; doi:10.1111/iju.70281)
Supplement: Supplementary file 1 — Table S1: List of investigators. Table S2: Best overall response by metastatic site and histology. Table S3: Time to onset and resolution of treatment‐related adverse events. Table S4: Patterns of treatment‐related adverse event management. Table S5: Baseline characteristics of patients who discontinued nivolumab‐plus‐ipilimumab due to AEs. Table S6: Baseline characteristics of patients who initiated second‐line treatment. Figure S1: Study design. Figure S2: Patient disposition. [file IJU-33-0-s001.docx]

# **Supporting information**

Table S1 List of investigators

Table S2 Best overall response by metastatic site and histology

Table S3 Time to onset and resolution of treatment-related adverse events

Table S4 Patterns of treatment-related adverse event management

Table S5 Baseline characteristics of patients who discontinued nivolumab-plus-ipilimumab due to AEs

Table S6 Baseline characteristics of patients who initiated second-line treatment

Figure S1 Study design

Figure S2 Patient disposition

## **Table S1 List of investigators**

| Site name | Investigator |
| --- | --- |
| Sapporo Medical University Hospital | Naoya Masumori |
| Hokkaido University Hospital | Takashige Abe |
| Hirosaki University Hospital | Chikara Ohyama |
| Iwate Medical University Hospital | Wataru Obara |
| Yamagata University Hospital | Norihiko Tsuchiya |
| Akita University Hospital | Kazuyuki Numakura |
| University of Tsukuba Hospital | Hiroyuki Nishiyama |
| Saitama Medical University International Medical Center | Suguru Shirotake |
| Chiba Cancer Center | Atsushi Komaru |
| Chiba University Hospital | Tomohiko Ichikawa |
| Tokyo Women’s Medical University, Adachi Medical Center | Tsunenori Kondo |
| The Jikei University Hospital | Takahiro Kimura |
| Institute of Science Tokyo Hospital | Yasuhisa Fujii |
| Nippon Medical School Hospital | Yukihiro Kondo |
| Juntendo University Hospital | Shigeo Horie |
| The Cancer Institute Hospital of JFCR | Junji Yonese |
| Yokohama City University Hospital | Hisashi Hasumi |
| Kanagawa Cancer Center | Takeshi Kishida |
| Niigata University Medical & Dental Hospital | Yoshihiko Tomita |
| Toyama University Hospital | Hiroshi Kitamura |
| Kanazawa University Hospital | Atsushi Mizokami |
| Nagoya City University Hospital | Shuzo Hamamoto |
| Aichi Medical University Hospital | Naoto Sassa |
| Nara Medical University Hospital | Kiyohide Fujimoto |
| University Hospital, Kyoto Prefectural University of Medicine | Osamu Ukimura |
| Osaka International Cancer Institute | Kazuo Nishimura |
| Osaka University Hospital | Norio Nonomura |
| Kindai University Hospital | Hirotsugu Uemura |
| Kobe University Hospital | Tomoaki Terakawa |
| Hiroshima University Hospital | Nobuyuki Hinata |
| Okayama University Hospital | Satoshi Katayama |
| Yamaguchi University Hospital | Koji Shiraishi |
| Tokushima University Hospital | Masayuki Takahashi |
| Kagawa University Hospital | Mikio Sugimoto |
| Ehime University Hospital | Takashi Saika |
| Kyushu University Hospital | Masatoshi Eto |
| Nagasaki University Hospital | Kojiro Ohba |

## **Table S2 Best overall response by metastatic site and histology**

|  | Metastasis site ^a^ | | | | |  | Histology | |
| --- | --- | --- | --- | --- | --- | --- | --- | --- |
|  | Lung  n = 171 | Lymph node  n = 119 | Bone  n = 82 | Liver  n = 37 | Brain  n = 7 |  | ccRCC  n = 224 | nccRCC  n = 50 |
| With measurable disease ^b^, n | 159 | 111 | 76 | 37 | 7 |  | 205 | 45 |
| BOR, n (%) |  |  |  |  |  |  |  |  |
| CR | 7 (4.4) | 4 (3.6) | 1 (1.3) | 2 (5.4) | 0 (0.0) |  | 15 (7.3) | 1 (2.2) |
| PR | 56 (35.2) | 43 (38.7) | 17 (22.4) | 12 (32.4) | 2 (28.6) |  | 64 (31.2) | 14 (31.1) |
| SD | 38 (23.9) | 26 (23.4) | 26 (34.2) | 4 (10.8) | 2 (28.6) |  | 58 (28.3) | 12 (26.7) |
| PD | 39 (24.5) | 25 (22.5) | 21 (27.6) | 13 (35.1) | 2 (28.6) |  | 43 (21.0) | 16 (35.6) |
| NE ^c^ | 19 (11.9) | 13 (11.7) | 11 (14.5) | 6 (16.2) | 1 (14.3) |  | 25 (12.2) | 2 (4.4) |
| ORR, n (%) | 63 (39.6) | 47 (42.3) | 18 (23.7) | 14 (37.8) | 2 (28.6) |  | 79 (38.5) | 15 (33.3) |
| 95% CI | 32.0–47.7 | 33.0–52.1 | 14.7–34.8 | 22.5–55.2 | 3.7–71.0 |  | 31.8–45.6 | 20.0–49.0 |
| DCR, n (%) | 101 (63.5) | 73 (65.8) | 44 (57.9) | 18 (48.6) | 4 (57.1) |  | 137 (66.8) | 27 (60.0) |
| 95% CI | 55.5–71.0 | 56.2–74.5 | 46.0–69.1 | 31.9–65.6 | 18.4–90.1 |  | 59.9–73.2 | 44.3–74.3 |

^a^ Each metastasis site included cases with multiorgan involvement. ^b^ Data were analyzed in patients with measurable diseases, assessed by investigators according to RECIST v1.1. PD was also determined by clinical progression. The number of patients with measurable disease served as the denominator for calculating the BOR, ORR, and DCR. ^c^ NE included patients for whom the assessment data were not available. BOR, best overall response; ccRCC, clear cell renal cell carcinoma; CI, confidence interval; CR, complete response; DCR, disease control rate; nccRCC, non-clear cell renal cell carcinoma; NE, not evaluable; ORR, objective response rate; PD, progressive disease; PR, partial response; RECIST, Response Evaluation Criteria in Solid Tumors; SD, stable disease.

## **Table S3 Time to onset and resolution of treatment-related adverse events**

|  | No. of patients,  n (%) | No. of events,  n | Median onset ^a^  (weeks) | No. of resolution,  n (%) | Median resolution ^b^  (weeks) |
| --- | --- | --- | --- | --- | --- |
| All TRAEs | 212 (77.4) | 508 | 7.1 | 410 (80.7) | 8.0 |
| Skin | 97 (35.4) | 110 | 4.6 | 92 (83.6) | 9.0 |
| Endocrine | 84 (30.7) | 110 | 8.9 | 73 (66.4) | 8.0 |
| Hepatic | 44 (16.1) | 47 | 6.3 | 43 (91.5) | 10.1 |
| Respiratory | 24 (8.8) | 24 | 6.6 | 23 (95.8) | 9.1 |
| Diarrhea/colitis | 21 (7.7) | 21 | 7.0 | 21 (100.0) | 6.9 |
| Metabolism | 18 (6.6) | 20 | 11.1 | 15 (75.0) | 2.6 |
| Gastrointestinal | 16 (5.8) | 24 | 9.8 | 19 (79.2) | 17.6 |
| Renal | 15 (5.5) | 15 | 6.3 | 10 (66.7) | 7.6 |
| Nervous | 15 (5.5) | 15 | 6.1 | 14 (93.3) | 4.7 |
| Musculoskeletal | 15 (5.5) | 15 | 14.4 | 12 (80.0) | 37.9 |
| Cardiovascular | 11 (4.0) | 13 | 7.4 | 10 (76.9) | 9.0 |
| Hematotoxicity | 8 (2.9) | 11 | 9.0 | 8 (72.7) | 5.0 |
| Acute pancreatitis | 4 (1.5) | 4 | 18.7 | 4 (100.0) | 29.5 |
| Increased amylase and lipase levels | 3 (1.1) | 4 | 6.4 | 4 (100.0) | 11.5 |
| Others | 52 (19.0) | 75 | 6.0 | 62 (82.7) | 3.1 |

^a^ Median onset was defined as the median time from the initiation of NIVO+IPI to the onset of TRAEs. ^b^ Median resolution was defined as the median resolution time from the onset of TRAEs to the date of resolution. Resolution included recovered, recovering, and recovered but with sequelae. NIVO+IPI, nivolumab-plus-ipilimumab; TRAE, treatment-related adverse event.

## **Table S4 Patterns of treatment-related adverse event management**

|  | All | Steroid | | | |  | Immunosuppressant agent | |
| --- | --- | --- | --- | --- | --- | --- | --- | --- |
|  |  | Total ^a^ | Low-dose | High-dose ^b^ | Pulse ^c^ |  | MMF | Infliximab |
| No. of patients, (%) | 274 | 164 (59.9) | 160 (58.4) | 54 (19.7) | 12 (4.4) |  | 2 (0.7) | 0 (0) |
| No. of events, (%) | 508 | 238 (46.9) | 231 (45.5) | 60 (11.8) | 12 (2.4) |  | 2 (0.4) | 0 (0) |
| Endocrine | 110 | 56 (50.9) | 56 (50.9) | 8 (7.3) | 1 (0.9) |  | 0 (0) | 0 (0) |
| Skin | 110 | 90 (81.8) | 89 (80.9) | 5 (4.5) | 2 (1.8) |  | 0 (0) | 0 (0) |
| Hepatic | 47 | 20 (42.6) | 18 (38.3) | 15 (31.9) | 3 (6.4) |  | 2 (4.3) | 0 (0) |
| Gastrointestinal | 24 | 9 (37.5) | 9 (37.5) | 4 (16.7) | 0 (0) |  | 0 (0) | 0 (0) |
| Respiratory | 24 | 11 (45.8) | 11 (45.8) | 6 (25.0) | 2 (8.3) |  | 0 (0) | 0 (0) |
| Diarrhea/colitis | 21 | 11 (52.4) | 11 (52.4) | 4 (19.0) | 0 (0) |  | 0 (0) | 0 (0) |
| Metabolism | 20 | 2 (10.0) | 2 (10.0) | 0 (0) | 0 (0) |  | 0 (0) | 0 (0) |
| Nervous | 15 | 3 (20.0) | 3 (20.0) | 2 (13.3) | 2 (13.3) |  | 0 (0) | 0 (0) |
| Musculoskeletal | 15 | 10 (66.7) | 10 (66.7) | 2 (13.3) | 0 (0) |  | 0 (0) | 0 (0) |
| Renal | 15 | 8 (53.3) | 7 (46.7) | 6 (40.0) | 0 (0) |  | 0 (0) | 0 (0) |
| Cardiovascular | 13 | 2 (15.4) | 1 (7.7) | 1 (7.7) | 1 (7.7) |  | 0 (0) | 0 (0) |
| Hematotoxicity | 11 | 3 (27.3) | 2 (18.2) | 3 (27.3) | 0 (0) |  | 0 (0) | 0 (0) |
| Increased amylase and lipase levels | 4 | 0 (0) | 0 (0) | 0 (0) | 0 (0) |  | 0 (0) | 0 (0) |
| Acute pancreatitis | 4 | 3 (75.0) | 3 (75.0) | 1 (25.0) | 0 (0) |  | 0 (0) | 0 (0) |
| Others | 75 | 10 (13.3) | 9 (12.0) | 3 (4.0) | 1 (1.3) |  | 0 (0) | 0 (0) |

^a^ Some patients used low- and high-dose steroids for an event. ^b^ High-dose steroids were defined as ≥ 40 mg per day of prednisone-equivalent. ^c^ Steroid pulse treatment was defined as ≥ 500 mg per day of prednisone-equivalent. MMF, mycophenolate mofetil.

## **Table S5 Baseline characteristics of patients who discontinued nivolumab-plus-ipilimumab due to AEs**

|  | Discontinued due to AEs |
| --- | --- |
|  | n = 86 |
| Male, n (%) | 71 (82.6) |
| Median age, years (range) | 69 (31–87) |
| ≥ 75 years, n (%) | 25 (29.1) |
| BMI ^a^, kg/m^2^, n (%) |  |
| < 18.5 | 11 (12.9) |
| ≥ 18.5, < 25.0 | 46 (54.1) |
| ≥ 25.0 | 28 (32.9) |
| Smoking status, n (%) |  |
| Never | 23 (26.7) |
| Past | 36 (41.9) |
| Current | 18 (20.9) |
| Unknown | 9 (10.5) |
| ECOG PS, n (%) |  |
| 0 | 60 (69.8) |
| 1 | 14 (16.3) |
| 2 | 9 (10.5) |
| ≥ 3 | 3 (3.5) |
| IMDC risk, n (%) |  |
| Intermediate | 52 (60.5) |
| Poor | 34 (39.5) |
| Histology, n (%) |  |
| ccRCC | 69 (80.2) |
| nccRCC | 17 (19.8) |
| With measurable disease ^b^, n (%) | 77 (89.5) |
| With primary tumor, n (%) | 40 (46.5) |
| With sarcoma component, n (%) | 13 (15.1) |
| Previous surgery, n (%) | 47 (54.7) |
| Previous nephrectomy, n (%) | 46 (53.5) |
| Radical nephrectomy, n (%) | 36 (41.9) |
| Previous radiation therapy, n (%) | 10 (11.6) |
| Number of metastatic organs, n (%) |  |
| 0 | 2 (2.3) |
| 1 | 38 (44.2) |
| 2 | 28 (32.6) |
| 3 | 11 (12.8) |
| ≥ 4 | 7 (8.1) |
| Site of metastasis, n (%) |  |
| Lung | 53 (61.6) |
| Lymph node | 39 (45.3) |
| Bone | 25 (29.1) |
| Liver | 10 (11.6) |
| Brain | 2 (2.3) |

^a^ Among the 86 patients who discontinued treatment due to AEs, one patient had missing BMI data.

^b^ Measurable disease was assessed by investigators using RECIST v1.1.

BMI, body mass index; ccRCC, clear cell renal cell carcinoma; ECOG PS, Eastern Cooperative Oncology Group performance status; IMDC, International Metastatic Renal Cell Carcinoma Database Consortium; nccRCC, non-clear cell renal cell carcinoma; RECIST, Response Evaluation Criteria in Solid Tumors.

## **Table S6 Baseline characteristics of patients who initiated second-line treatment**

|  | Initiating 2L treatment ^a^ |
| --- | --- |
|  | n = 132 |
| Male, n (%) | 102 (77.3) |
| Median age, years (range) | 67 (32–87) |
| ≥ 75 years, n (%) | 23 (17.4) |
| BMI ^b^, kg/m^2^, n (%) |  |
| < 18.5 | 15 (11.5) |
| ≥ 18.5, < 25.0 | 77 (58.8) |
| ≥ 25.0 | 39 (29.8) |
| Smoking status, n (%) |  |
| Never | 38 (28.8) |
| Past | 51 (38.6) |
| Current | 30 (22.7) |
| Unknown | 13 (9.8) |
| ECOG PS, n (%) |  |
| 0 | 95 (72.0) |
| 1 | 20 (15.2) |
| 2 | 16 (12.1) |
| ≥ 3 | 1 (0.8) |
| IMDC risk, n (%) |  |
| Intermediate | 78 (59.1) |
| Poor | 54 (40.9) |
| Histology, n (%) |  |
| ccRCC | 108 (81.8) |
| nccRCC | 24 (18.2) |
| With measurable disease ^c^, n (%) | 126 (95.5) |
| With primary tumor, n (%) | 74 (56.1) |
| With sarcoma component, n (%) | 10 (7.6) |
| Previous surgery, n (%) | 62 (47.0) |
| Previous nephrectomy, n (%) | 60 (45.5) |
| Radical nephrectomy, n (%) | 48 (36.4) |
| Previous radiation therapy, n (%) | 12 (9.1) |
| Number of metastatic organs, n (%) |  |
| 0 | 3 (2.3) |
| 1 | 51 (38.6) |
| 2 | 49 (37.1) |
| 3 | 19 (14.4) |
| ≥ 4 | 10 (7.6) |
| Site of metastasis, n (%) |  |
| Lung | 91 (68.9) |
| Lymph node | 56 (42.4) |
| Bone | 38 (28.8) |
| Liver | 20 (15.2) |
| Brain | 1 (0.8) |

^a^ Among the 132 patients who initiated the 2L treatment, 38 (28.8%) discontinued NIVO+IPI due to AEs.

^b^ Among the 132 patients who initiated the 2L treatment, one had missing BMI data. ^c^ Measurable disease was assessed by investigators using RECIST v1.1.

AE, adverse event; BMI, body mass index; ccRCC, clear cell renal cell carcinoma; ECOG PS, Eastern Cooperative Oncology Group performance status; IMDC, International Metastatic Renal Cell Carcinoma Database Consortium; nccRCC, non-clear cell renal cell carcinoma; NIVO+IPI, nivolumab-plus-ipilimumab; 2L, second-line; RECIST, Response Evaluation Criteria in Solid Tumors.

## **Figure S1 Study design**


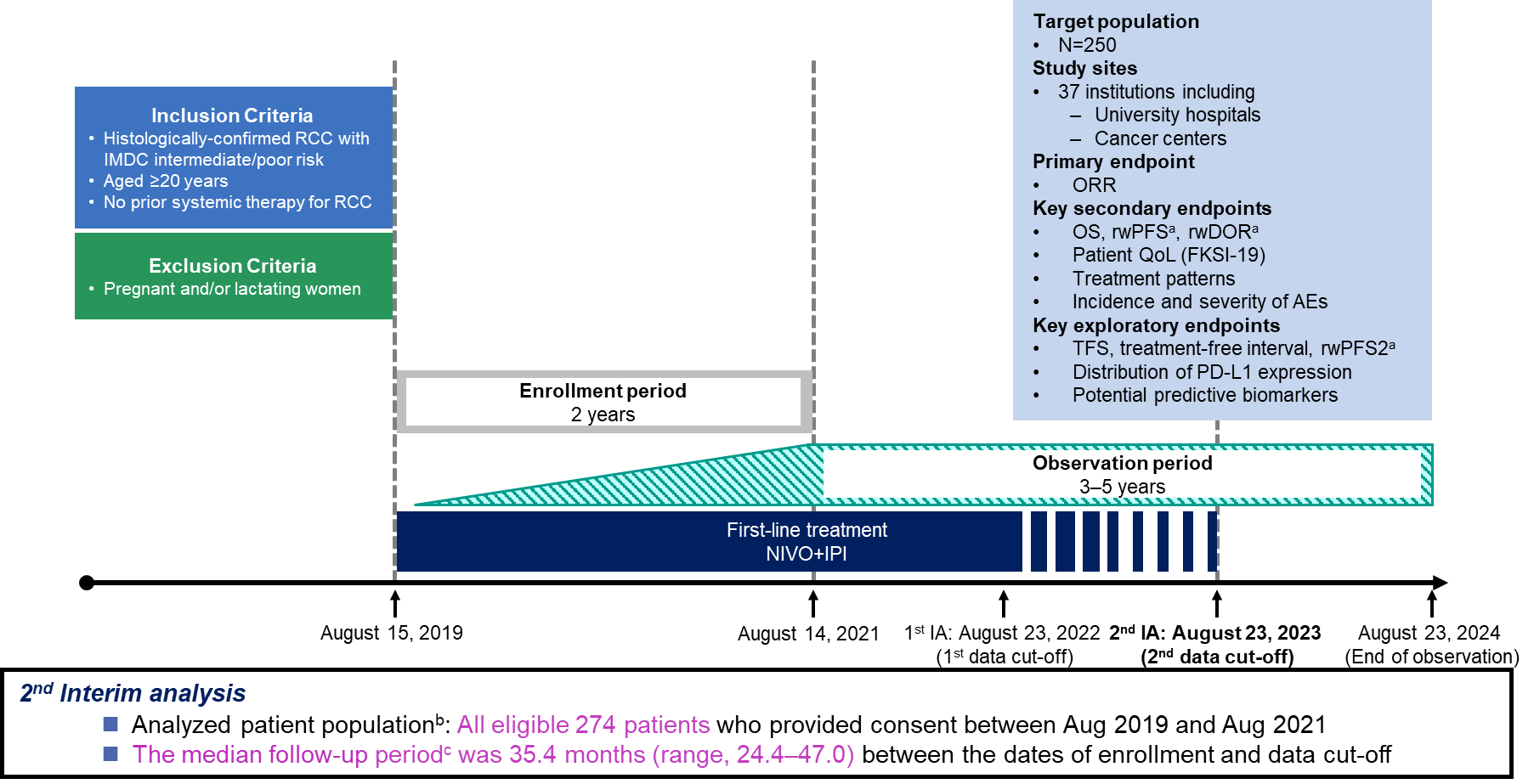


^a^ Regarding rwPFS, rwDOR, and rwPFS2, although the first interim report of J-ENCORE^16^ defined them as PFS, DOR, and PFS2, we designated the terms, rwPFS, rwDOR, and rwPFS2, to highlight that these endpoints were derived from real-world clinical practice. The definitions of rwPFS, rwDOR, and rwPFS2 in this second interim report are identical to those of PFS, DOR, and PFS2 in the first interim report.^16^ ^b^ For the 2nd interim analysis, the data were cut off on August 23, 2023, and the database was locked on March 13, 2024. Some data obtained after data cut-off, including safety information, were entered into the database. ^c^ The follow-up period was defined as the period between the dates of enrollment and data cut-off. AE, adverse event; FKSI-19, functional assessment of cancer therapy-kidney symptom index 19; IA, interim analysis; IMDC, International Metastatic Renal Cell Carcinoma Database Consortium; NIVO+IPI, nivolumab-plus-ipilimumab; ORR, objective response rate; OS, overall survival; PD-L1, programmed cell death ligand 1; QoL, quality of life; RCC, renal cell carcinoma; rwDOR, real-world duration of response; rwPFS, real-world progression-free survival; rwPFS2, real-world PFS after initiation of second-line treatment; TFS, treatment free survival.

## **Figure S2** **Patient disposition**


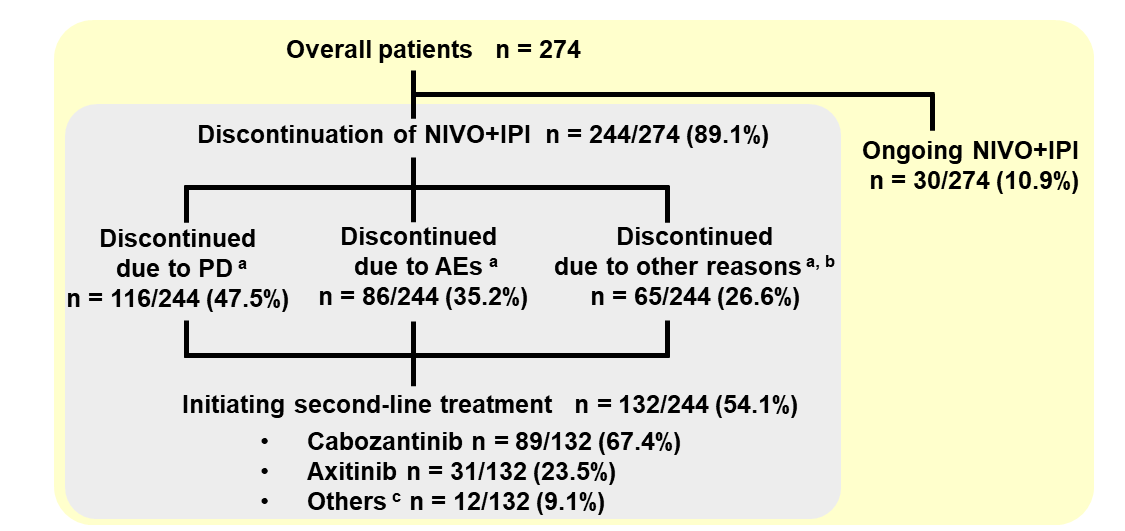


^a^ Multiple answers were allowed for reasons to discontinue NIVO+IPI. ^b^ Only reasons for NIVO discontinuation were counted. Other reasons included good response, transfer to other hospital, personal reason, death, and others. ^c^ Others included pazopanib, sunitinib, nivolumab, and sorafenib. AEs, adverse events; NIVO+IPI, nivolumab-plus-ipilimumab; PD, progressive disease.
